# A Method for Measuring the Height of Hand Movements Based on a Planar Array of Electrostatic Induction Electrodes

**DOI:** 10.3390/s20102943

**Published:** 2020-05-22

**Authors:** Linyi Zhang, Xi Chen, Pengfei Li, Chuang Wang, Mengxuan Li

**Affiliations:** State Key Laboratory of Mechatronics Engineering and Control, Beijing Institute of Technology, Beijing 100081, China; zhang_linyi@foxmail.com (L.Z.); pfli@bit.edu.cn (P.L.); cunghens@163.com (C.W.); formlmx@126.com (M.L.)

**Keywords:** the height of hand movements, non-contact HCI, electrostatics field

## Abstract

This paper proposes a method based on a planar array of electrostatic induction electrodes, which uses human body electrostatics to measure the height of hand movements. The human body is electrostatically charged for a variety of reasons. In the process of a hand movement, the change of a human body’s electric field is captured through the electrostatic sensors connected to the electrode array. A measurement algorithm for the height of hand movements is used to measure the height of hand movements after the direction of it has been obtained. Compared with the tridimensional array, the planar array has the advantages of less space and easy deployment; therefore, it is more widely used. In this paper, a human hand movement sensing system based on human body electrostatics was established to perform verification experiments. The results show that this method can measure the height of hand movements with good accuracy to meet the requirements of non-contact human-computer interactions.

## 1. Introduction

The progress of computer and network technology and the continuous expansion of its application fields have promoted the continuous evolution of human-computer interactions. Determining how to interact with various smart devices conveniently and effectively is one of the hot topics of research today. As a new generation of human-computer interaction, the natural user interface (NUI) [1] is gradually replacing the command-line and graphical user interfaces represented by the keyboard and the mouse [2,3].

The NUI does not require users to learn pre-designed operations but interacts with computers through voice, hand movements, facial expressions, or body gestures. Compared with voice, hand movement-based interaction has the advantage of being cross-cultural, and is not easily affected by background noise [4]; compared with facial expressions and body gestures, hand movement-based interaction is more comfortable, and users do not become fatigued after long-term use [5]. At present, hand movement-based interaction systems are generally divided into two types, namely, non-wearable device-based interaction methods and wearable device-based interaction methods [6]. Vision-based interaction [7] is a typical non-wearable device-based interaction, which can realize non-contact human-computer interactions, but its algorithm is complex and it is easily affected by ambient light, background, clothing, and so on [8]. In addition, this interaction relies on the exposed camera to obtain the user’s gesture information, which not only destroys the design sense of the devices but also causes privacy issues for the user [9]. Wearable device-based methods have high detection accuracy [10], but these methods require users to wear devices. For example, wearable electromyography (EMG)-based interaction requires electrodes to be stuck onto the forearm or wrist, which is not suitable in some scenarios [11,12]. Therefore, a new type of human-computer interaction method with good user experience and avoiding privacy problems will be of practical significance.

In recent years, due to the development of human body electrostatic detection technology [13,14], research on human-computer interactions based on human body static electricity has gradually risen [15,16] and has attracted widespread attention. The human body is electrostatically charged in certain environments and for various reasons. When the hand moves, the electrostatic charge of the hand disturbs the electric field of the space. Electrostatic induction on the electrode caused by the disturbance can be sensed by electrostatic sensors so that the parameters of a hand movement can be obtained to realize human-computer interactions. This detection method has the advantages of non-contact, good privacy, simple algorithm, and low power consumption [17]. In 2007, Professor Takiguchi of Japan proposed to use electrodes to detect electrostatic signals generated by human walking, which has the characteristics of a low false alarm rate, small dead area, and simple system design and deployment compared to other human detection technologies [18,19]. Kai Tang used a tridimensional array of electrodes to measure the direction [20] and speed of hand movements and proposed a real-time hand position sensing technology based on human body electrostatics for human-computer interactions [21]. Both of these methods need a tridimensional array of electrodes, which cannot adapt to scenarios where the placement or operating space is narrow, but the height of a hand movement is needed.

Kurita proposed a non-contact electrostatics detection method to detect human foot movements and applied it to throwing movement analysis [22,23,24]. A planar array of four detection electrodes was used to classify hand movements in eight directions of the plane [16]. In some interaction scenarios, direction information alone is not enough to support the amount of information needed for interaction.

In this paper, a method for measuring the height of hand movements based on a planar array of electrostatic induction electrodes is proposed. This method uses the measurement algorithm for the height of hand movements based on human body electrostatics to calculate the distance between the trajectory of the hand movement and the electrode array. The method can be applied to non-contact human-computer interactions. A planar array of electrostatic induction electrodes was used to obtain not only the direction but also the height of hand movements, which can meet the requirements of human-computer interactions under various space conditions.

## 2. Methods

The human body has a certain charge due to friction and contact with other objects [25], which is called the human body electrostatics. As the tip of the charged human body, the hand changes the distribution of the electric field in space when it moves, which leads to the flow of the induced charge on the electrode, that is, the induced current [16].

**Figure 1:**
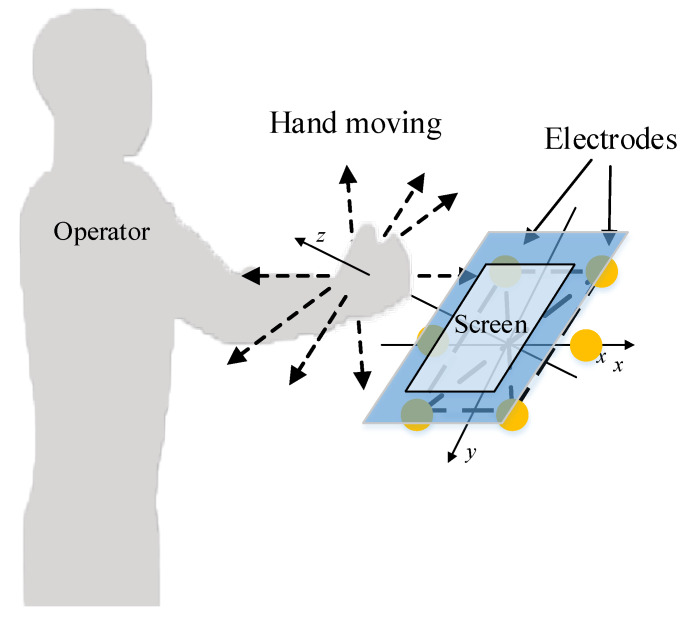
Schematic diagram of the interaction.

The previous method only focused on the zero-crossing point and the maximum point of the induced current signal without discussing the moment when the maximum point would appear. As a result, only the direction of the hand movement on the two-dimensional plane can be obtained. In this paper, it is proposed to calculate the occurrence time of maximum points, zero-crossing points, and the change rate’s maximum points of the induced signals on multiple electrodes transmitted to the upper computer. On the basis of judging the direction of a hand movement, the upper computer measures the height of the hand movement and then recognizes the interactive intention expressed by the hand trajectory to realize a non-contact human-computer interaction. The schematic diagram of the interaction is shown in Figure 1.

When the hand moves linearly in different directions in front of the array, the time when the zero-crossing points of the induced current are generated on the electrodes at different positions is different. By processing the signals on each electrode, the hand movement in eight directions can be classified, which is presented in Reference [16]. The following sections will introduce, in detail, the measurement algorithm for the height of hand movements based on human body electrostatics.

### 2.1. Regularity of the Occurrence of the Maximum Value of the Induced Current, the Zero-Crossing of the Induced Current, and the Maximum Value of the Change Rate of the Induced Current

When the hand moves linearly near the spherical electrode, an induced current is generated on it. Suppose that the hand charge quantity is *Q*, the distance between the trajectory of the hand movement and electrode is *z*_0_, and the velocity of the hand is *v* for a very short time when the hand moves near the electrode.

**Figure 2:**
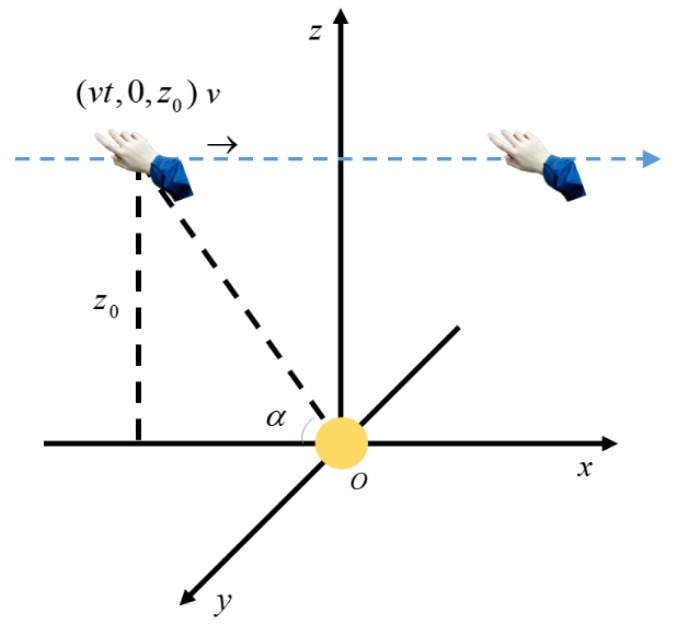
Schematic diagram of a hand movement in a straight line near the spherical electrode.

The left-hand coordinate system is established with the ball center as the origin. For the convenience of calculation, let the trajectory of the hand movement be in the *XOZ* plane and parallel to the *x*-axis, as shown in Figure 2. Take the time when the hand is on the *z*-axis as time zero, and the angle between the *x*-axis and the line passing through the hand and the origin is *α*.

The induced current signal of the spherical electrode can be expressed as:

(1) $$i=\frac{QR_{0}}{r^{2}}\frac{dr}{dt}=QR_{0}\cdot \frac{v^{2}t}{{\left[{\left(vt\right)}^{2}+z_{0}{}^{2}\right]}^{\frac{3}{2}}}$$

where *R*_0_ is the radius of the spherical electrode, and *r* is the distance from the trajectory of the hand to the center of the spherical electrode.

In addition, the *α* can be calculated as:

(2) $$\cos\alpha =\sqrt{\frac{v^{2}t^{2}}{\left[z_{0}{}^{2}+{\left(-vt\right)}^{2}\right]}}.$$

**Figure 3:**
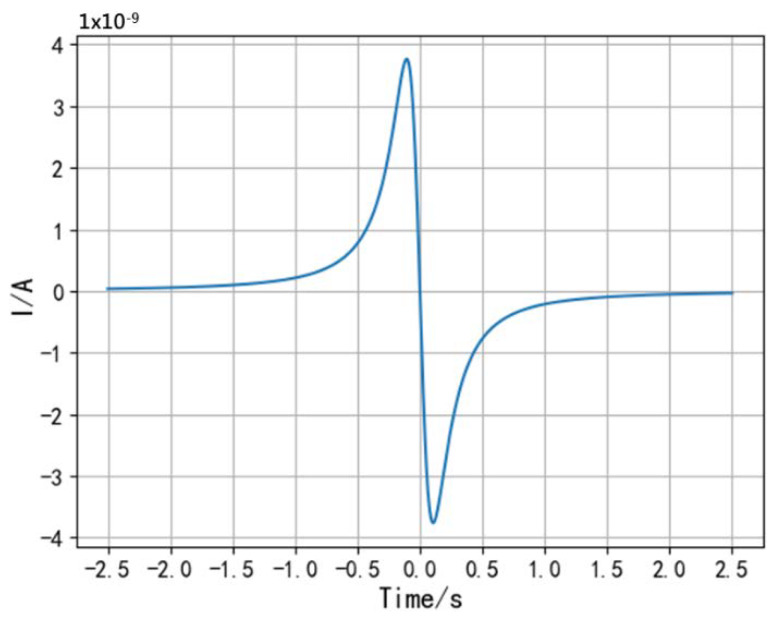
Waveform of the induced current.

Take in the parameters (set *v* = 2 m/s, *z*_0_ = 0.3 m, *Q* = −10^−9^ C, *R*_0_ = 0.004 m) to simulate the current *i*, and the current waveform is shown in Figure 3.

The waveform of the induced current *i* in the figure has two extreme points and one zero-crossing point. In order to calculate the position relationship between the hand and the electrode when the extreme points occur, differentiate the induced current *i* to *t* to obtain the change rate of the induced current as follows:

(3) $$i\prime =QR_{0}\cdot \frac{v^{2}}{{\left[{\left(vt\right)}^{2}+z_{0}{}^{2}\right]}^{\frac{3}{2}}}-QR_{0}\cdot \frac{3v^{4}t^{2}}{{\left[{\left(vt\right)}^{2}+z_{0}{}^{2}\right]}^{\frac{5}{2}}}.$$

**Figure 4:**
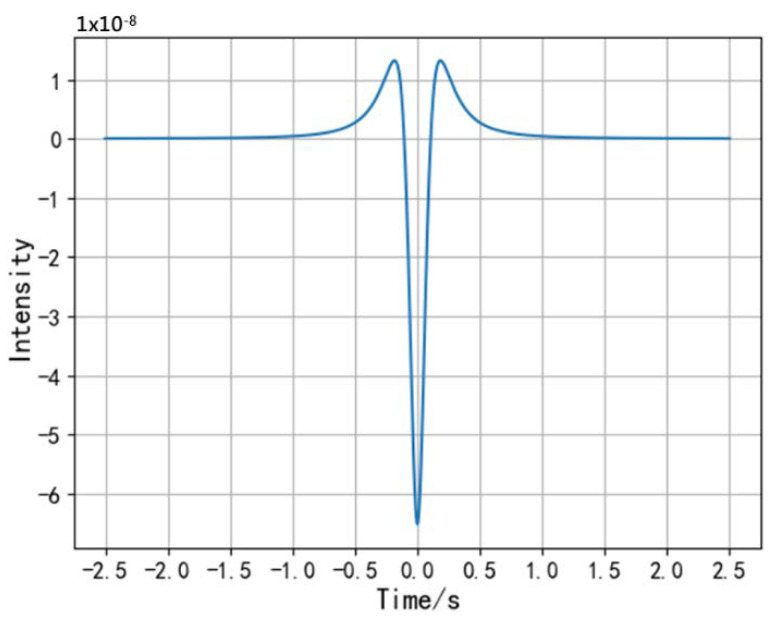
Waveform of the change rate of the induced current.

The simulated waveform of the change rate of the induced current $i^{\prime }$ is shown in Figure 4.

When the maximum value of the induced current *i* occurs, the change rate of the induced current is equal to zero, that is:

(4) $$i\prime =QR_{0}\cdot \frac{v^{2}}{{\left[{\left(vt\right)}^{2}+z_{0}{}^{2}\right]}^{\frac{3}{2}}}-QR_{0}\cdot \frac{3v^{4}t^{2}}{{\left[{\left(vt\right)}^{2}+z_{0}{}^{2}\right]}^{\frac{5}{2}}}=0.$$

So that:

(5) $$\cos\alpha =\sqrt{\frac{v^{2}t^{2}}{\left[z_{0}{}^{2}+{\left(vt\right)}^{2}\right]}}=\frac{\sqrt{3}}{3}.$$

That is, when *α* = 54.7°, the maximum value of the induced current *i* appears. The waveform of the change rate of the induced current has three extreme points. Similarly, let the second derivative of the induced current *i* to *t* be equal to zero.

(6) $$i^{\prime \prime }=QR_{0}\cdot \frac{-9v^{4}t}{{\left[z_{0}{}^{2}+{\left(-vt\right)}^{2}\right]}^{\frac{5}{2}}}+QR_{0}\cdot \frac{15v^{6}t^{3}}{{\left[z_{0}{}^{2}+{\left(-vt\right)}^{2}\right]}^{\frac{7}{2}}}=0.$$

So that:

(7) $$\cos\alpha =\sqrt{\frac{v^{2}t^{2}}{\left[z_{0}{}^{2}+{\left(-vt\right)}^{2}\right]}}=\sqrt{\frac{3}{5}}\;\mathrm{or}\;t=0.$$

When $\cos\alpha =\sqrt{\frac{3}{5}}$, that is, α = 39.2°, the first maximum value of the change rate of the induced current $i^{\prime }$ appears. When t=0, the hand is directly above the electrode, α_3_ = 90°, the minimum value of the change rate of the induced current $i^{\prime }$ appears, and the zero-crossing of the induced current *i* appears.

In summary, when α = 39.2°, the first maximum value of the change rate of the induced current $i^{\prime }$ appears; when α = 54.7°, the maximum value of the induced current *i* appears; and when α = 90°, the zero-crossing of the induced current *i* appears. For the convenience of the introduction later, let α_1_= 39.2°, α_2_ = 54.7°, and α_3_ = 90°.

### 2.2. Algorithm for Measuring the Distance between the Trajectory of a Hand Movement and the Straight Line of Two Electrodes

**Figure 5:**
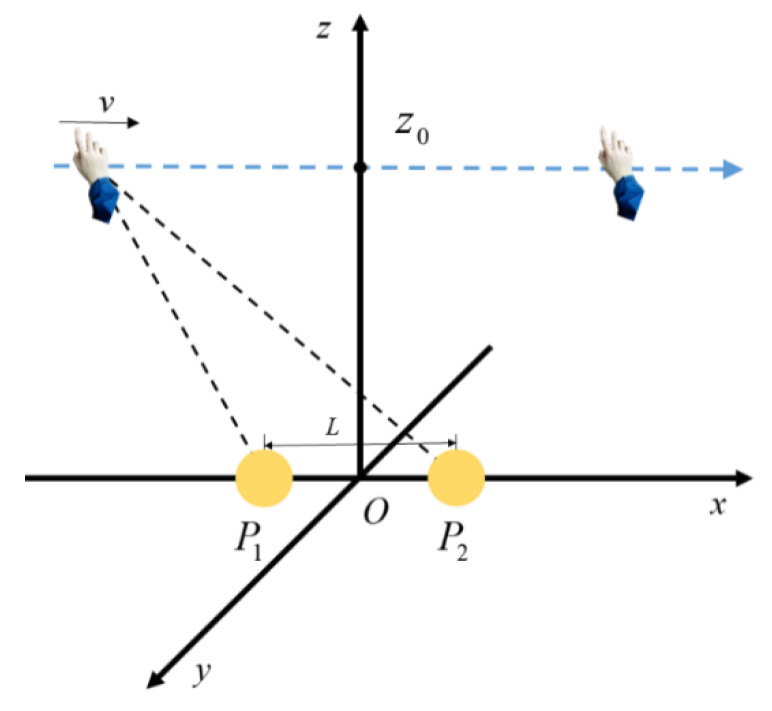
Schematic diagram of a hand movement parallel to the straight line of two electrodes.

Assume that the trajectory of a hand movement is parallel to the straight line of two electrodes; the distance between them can be calculated based on the values mentioned above. Take the midpoint of the connection of electrodes *P*_1_ and *P*_2_ as the origin, and the line of two electrodes is the *x*-axis. Set the trajectory of the hand movement in the *XOZ* plane and establish the coordinate system as shown in Figure 5. Set the electrode spacing as *L*.

**Figure 6:**
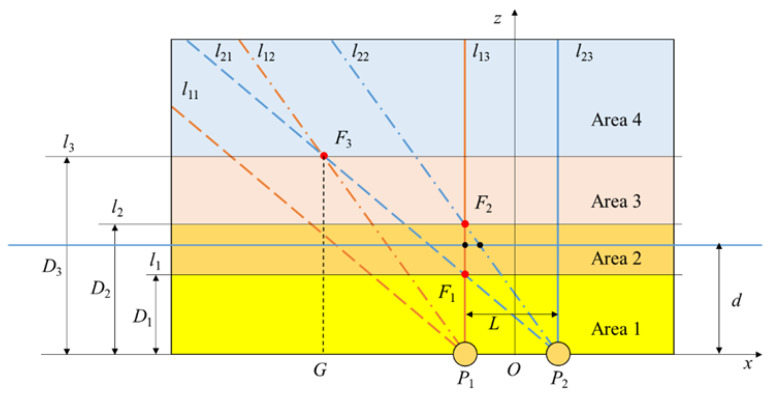
Schematic diagram of the area division above the electrodes.

According to the order of the maximum values of the induced current, the maximum values of the change rate of the induced current, and the zero-crossings of the induced current on the two electrodes, we can classify the range of the hand trajectory in the upper half of the *XOZ* plane, as shown in Figure 6.

In Figure 6, *P*_1_ and *P*_2_ are spherical electrodes placed on the *x*-axis. Make lines *l*_11_ and *l*_21_ have an angle of α_1_= 39.2° with the *x*-axis through *P*_1_ and *P*_2_. When the hand moves along the *x*-axis, the trajectory of the hand intersects with *l*_11_ and *l*_21_ successively, while the maximum values of the change rate of the induced current on *P*_1_ and *P*_2_ occur successively. Let their occurrence time be *T*_11_ and *T*_21_. Make lines *l*_12_ and *l*_22_ have an angle of α_2_ = 54.7° with the *x*-axis through *P*_1_ and *P*_2_. When the hand moves along the *x*-axis, the trajectory of the hand intersects with *l*_12_ and *l*_22_ successively, while the maximum values of the induced current on *P*_1_ and *P*_2_ occur successively. Let their occurrence time be *T*_12_ and *T*_22_. Make lines *l*_13_ and *l*_23_ have an angle of α_3_ = 90° with the *x*-axis through *P*_1_ and *P*_2_. When the hand moves along the *x*-axis, the trajectory of the hand intersects with *l*_13_ and *l*_23_ successively, while the zero-crossings of the induced current on *P*_1_ and *P*_2_ occur successively. Let their occurrence time be *T*_13_ and *T*_23_. *F*_1_, *F*_2_, and *F*_3_ are the intersections of *l*_13_ and *l*_21_, *l*_13_ and *l*_22_, and *l*_12_ and *l*_21_, respectively. *l*_1_, *l*_2_, and *l*_3_ are straight lines passing through *F*_1_, *F*_2_, and *F*_3_ and parallel to the *x*-axis. Their distances from the *x*-axis are *D*_1_, *D*_2_, and *D*_3_, respectively.

In the right-angled triangle *F*_1_*P*_1_*P*_2_ ∠ *F*_1_*P*_2_*P*_1_ = α_1_, *P*_2_*P*_1_ = *L*, so:

(8) $$\frac{D_{1}}{\tan\alpha_{1}}=L.$$

In the right-angled triangle *F*_2_*P*_1_*P*_2_, ∠ *F*_2_*P*_2_*P*_1_ = $\alpha_{2}$, *P*_2_*P*_1_ = *L*, so:

(9) $$\frac{D_{2}}{\tan\alpha_{2}}=L.$$

In the triangle *F*_3_*P*_1_*P*_2_, make the line segment *F*_3_*G* perpendicular to the *x*-axis, ∠*F*_3_*P*_2_*P*_1_ = α_1_, ∠*F*_3_*P*_2_*P*_1_ = α_2_, *P*_2_*P*_1_ = *L*, so:

(10) $$\frac{D_{3}}{\tan\alpha_{1}}-\frac{D_{3}}{\tan\alpha_{2}}=L.$$

It can be calculated that:

(11) $$\left\{ \begin{array}{l} D_{1}=\frac{\sqrt{6}}{3}L\\ D_{2}=\sqrt{2}L\\ D_{3}=(\frac{\sqrt{6}+\sqrt{2})}{2}L \end{array}\right..$$

*l*_1_, *l*_2_, and *l*_3_ divide the upper part of the *x*-axis into four areas of different heights. For convenience, they are named “Area 1”, “Area 2”, “Area 3”, and “Area 4”.

When the trajectory of a hand movement is located in different areas above the electrode, the occurrence time of the maximum values of the induced current, the zero-crossings of the induced current, and the maximum values of the change rate of the induced current on the two electrodes are different. Take the trajectory of the hand movement in Areas 3 and 2 as examples. When the hand moves along the *x*-axis, the trajectory intersects with *l*_11_, *l*_12_, *l*_21_, *l*_22_, *l*_13_, and *l*_23_ successively if it is in Area 3; in this case, *T*_11_ < *T*_12_ < *T*_21_ < *T*_22_ < *T*_13_ < *T*_23_. When the hand moves along the *x*-axis, the trajectory intersects with *l*_11_, *l*_12_, *l*_21_, *l*_13_, *l*_22,_ and *l*_23_ successively if it is in Area 2. The difference is, in this case, *T*_11_ < *T*_12_ < *T*_21_ < *T*_13_ < *T*_22_ < *T*_23_. By sorting the six moments, the position of the trajectory of a hand movement parallel to the *x*-axis is divided into four areas. Based on this, the distance *d* between the trajectory of the hand movement and the *x*-axis can be calculated by the following method.

Suppose that the hand moves along the *x*-axis; no matter which area the trajectory is located in, it passes through *l*_13_ first, and then *l*_23_, after the time difference *Δt*_0_. In the very short time that the hand passes the electrode, the hand movement speed *v* can be regarded as a fixed value:

(12) $$v=\frac{L}{\Delta t_{0}}.$$

Taking the height *D*_2_ as a reference, let the time difference between the hands passing *l*_13_ and *l*_22_ be *Δt*_2_ (when *d* < *D*_2_, *Δt*_2_ is positive; when *d* = *D*_2_, *Δt*_2_ is zero; when *d* > *D*_2_, *Δt*_2_ is negative), then *d* can be calculated as:

(13) $$d=D_{2}-\sqrt{2}v\Delta t_{2}.$$

Substituting Equation (12) into Equation (13):

(14) $$d=D_{2}-\sqrt{2}L\frac{\Delta t_{2}}{\Delta t_{0}}.$$

The distance *d* between the trajectory of a hand movement and the *x*-axis can be calculated through Equation (14). In practical applications, in order to accurately calculate *d*, first determine the area in which the hand is located, and calculate *d* with one or more of *D*_1_, *D*_2_, and *D*_3_ as a reference.

If taking the height *D*_1_ as a reference, let the time difference between the hands passing *l*_13_ and *l*_21_ be *Δt*_1_ (when *d* < *D*_1_, *Δt*_1_ is positive; when *d* = *D*_1_, *Δt*_1_ is zero; when *d* > *D*_1_, *Δt*_1_ is negative), then *d* can be calculated as:

(15) $$d=D_{1}-\frac{\sqrt{6}}{3}v\Delta t_{1}=D_{1}-\frac{\sqrt{6}}{3}L\frac{\Delta t_{1}}{\Delta t_{0}}.$$

If taking the height *D*_3_ as a reference, let the time difference between the hands passing *l*_12_ and *l*_21_ be *Δt*_3_ (when *d* < *D*_3_, *Δt*_1_ is positive; when *d* = *D*_3_, *Δt*_3_ is zero; when *d* > *D*_3_, *Δt*_3_ is negative), then *d* can be calculated as:

(16) $$d=D_{3}-\frac{\sqrt{6}+\sqrt{2}}{2}v\Delta t_{3}=D_{3}-\frac{\sqrt{6}+\sqrt{2}}{2}L\frac{\Delta t_{3}}{\Delta t_{0}}.$$

In the actual application scenario, we can first determine the area where the hand is, then select the appropriate formula to calculate the value of *d*. If it is determined that the trajectory of the hand movement is in Area 1, calculate *d* by Equation (14); if it is determined that the trajectory of the hand movement is in Area 2, calculate *d* by Equations (14) and (15), respectively, and take their average value as the final result; if it is determined that the trajectory of the hand movement is in Area 3, calculate *d* by Equations (15) and (16), respectively, and take their average value as the final result; if it is determined that the trajectory of the hand movement is in Area 4, calculate *d* by Equation (16).

### 2.3. Measurement Algorithm for the Height of Hand Movements Based on Human Body Electrostatics

**Figure 7:**
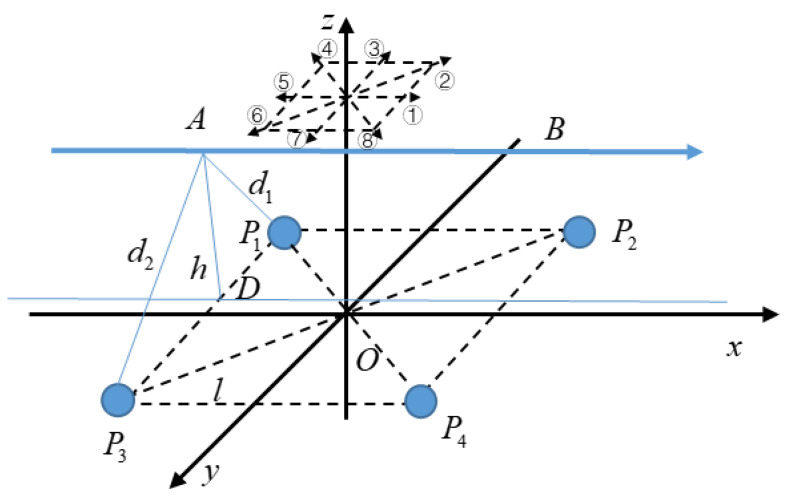
Schematic diagram of the height of a hand movement.

The height of a hand movement can be calculated by making up a planar array of multiple electrodes, that is, the vertical distance between the trajectory of the hand movement and the planar array of electrodes. Using an array of four spherical electrodes, the coordinate system as shown in Figure 7 is established by taking the midpoint of the square with four electrodes as the vertex as the origin. The electrodes *P*_1_, *P*_2_, *P*_3_, and *P*_4_ are all in the *XOY* plane, and the distance of each electrode from the *x* and *y* axes is *l*. The height of the hand movement is the distance *h* between the trajectory of the hand movement and the *XOY* plane.

For convenience, the eight directions on the plane are numbered in the order as shown in Figure 7. In most cases, the distance *d* measured by two electrodes is not equal to the height of the hand movement *h*. Take direction ① as an example. *AB* is the trajectory of the hand movement parallel to the *x*-axis, and *AD* is perpendicular to point *D* on *P*_1_*P*_3_. The length of segment *AD* is the height of the hand movement *h*. The distance measured by electrodes *P*_1_ and *P*_2_ between the trajectory and the straight line *P*_1_*P*_2_ is *d*_1_ and the distance measured by electrodes *P*_3_ and *P*_4_ between the trajectory and the straight line *P*_3_*P*_4_ is *d*_2_.

In the triangle *AP*_1_*P*_3_, the lengths of the three sides are *d*_1_, *d*_2_, and 2*l,* respectively. According to Heron’s formula, the area of the triangle *AP*_1_*P*_3_ is:

(17) $$S=\sqrt{p(p-d_{1})(p-d_{2})(p-2l)}$$

where *p* is the half perimeter of the triangle *AP*_1_*P*_3_, that is, *p* = (*d*_1_ + *d*_2_ + 2*l*)/2.

The value of the height of the hand movement can be calculated by:

(18) $$h=\frac{2S}{2l}=\frac{\sqrt{2d_{1}^{2}+2d_{2}^{2}-{\left(\frac{d_{1}^{2}-d_{2}^{2}}{2l}\right)}^{2}-4l^{2}}}{2}.$$

**Figure 8:**
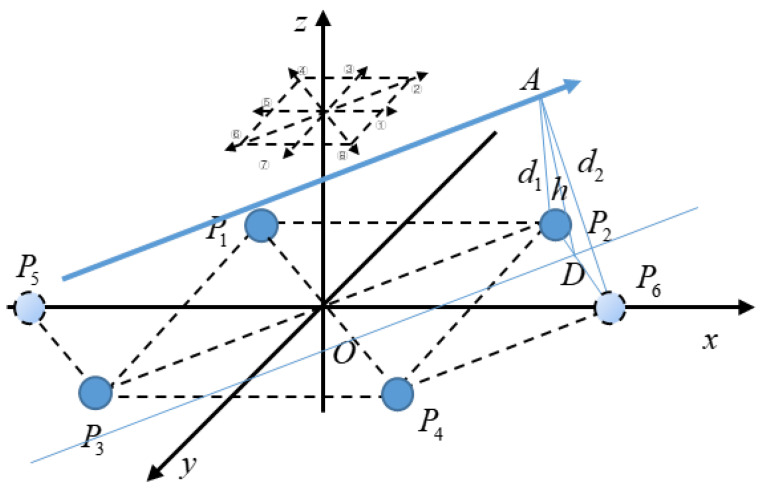
Schematic diagram of the electrode array improvement.

When the direction of hand movement is ②, ④, ⑥, or ⑧, there is only one set of electrodes in these directions, so there will be an error between the electrode measurement result *d* and the actual height *h*. This can be improved by adding electrodes, such as electrodes *P*_5_ and *P*_6_ shown in Figure 8, which are located on the *x*-axis, and *P*_4_*P*_6_ = *P*_3_*P*_5_ = *P*_2_*P*_6_ = *P*_1_*P*_5_ =$\sqrt{2}l$.

For example, when it is determined that the direction of the hand movement is direction ②, calculate the distance *d*_1_ from the hand trajectory to line *P*_2_*P*_3_ through Equation (14) by using the signals on electrodes *P*_2_ and *P*_3_. The distance between electrodes *P*_2_ and *P*_3_ is $L=2\sqrt{2}l$. Then, calculate the distance *d*_2_ from the hand trajectory to line *P*_4_*P*_6_ through Equation (15) by using the signals on electrodes *P*_4_ and *P*_6_. The distance between electrodes *P*_4_ and *P*_6_ is $L=\sqrt{2}l$, and the height of the hand movement *h* is:

(19) $$h=\frac{\sqrt{2d_{1}^{2}+2d_{2}^{2}-{\left(\frac{d_{1}^{2}-d_{2}^{2}}{\sqrt{2}l}\right)}^{2}-2l^{2}}}{2}.$$

**Figure 9:**
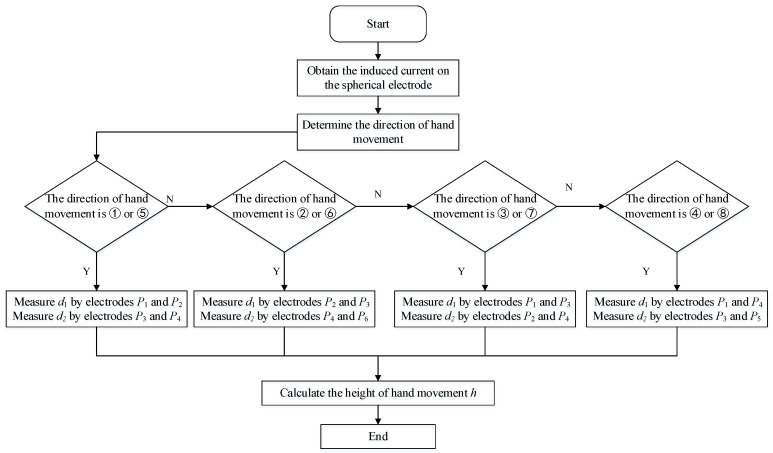
Flow chart for the hand height measurement.

The flowchart for obtaining the height of the hand movement is shown in Figure 9.

First, determine the direction of the hand movement and select the corresponding electrode. Then, by analyzing the order of the maximum values of the induced current, the maximum values of the change rate of the induced current, and the zero-crossings of the induced current on the two electrodes, we can know which area the hand is located in order to calculate *d*_1_ and *d*_2_.

### 2.4. Simulation Noise Test

**Figure 10:**
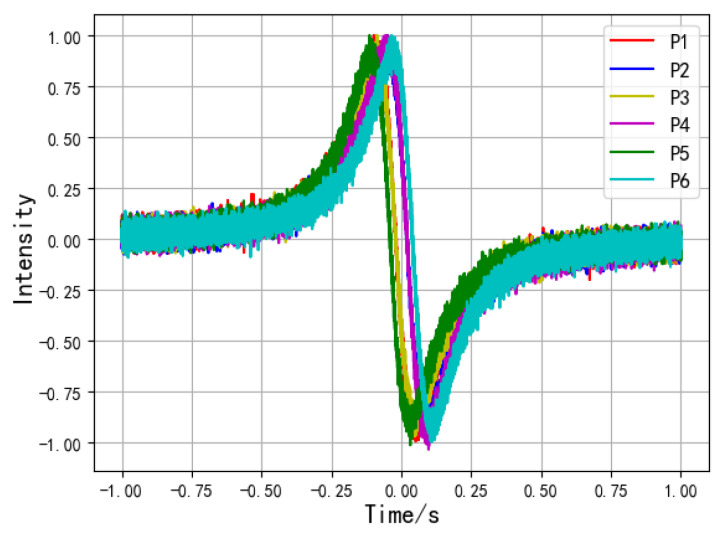
Simulation waveform of the induced current after adding noise.

To verify the practicability of the algorithm, white noise was applied to the induced current signals generated on the electrodes by hand movements of different heights. The simulated waveform of the induced current on the electrodes after adding noise is shown in Figure 10. In the figure, the current signals are normalized to −1~1.

The noise-added signals were used to calculate the height of the hand 100 times under different SNR (SIGNAL-NOISE RATIO) conditions. The absolute values of the mean errors and the maximum errors obtained are shown in Table 1.

It can be seen from the table that a low SNR affects the accuracy of the algorithm. With the SNR increased, the mean errors and the maximum errors are decreased. When the SNR is greater than 15 dB and the range is 50~350 mm, the maximum absolute error of the height measurement is 11.70 mm and the maximum relative error is 5.85%.

### 2.5. Design and Experiment 

**Figure 11:**
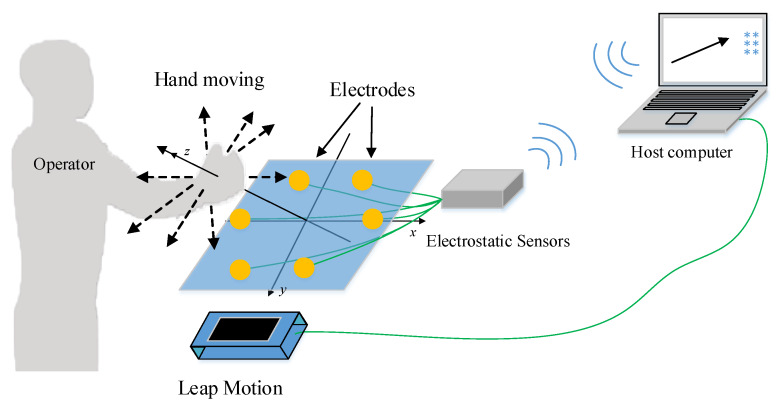
Schematic diagram of the verification system.

In order to verify the algorithm proposed in this paper, we designed a human hand movement sensing system based on human body electrostatics, including a planar array of four spherical electrodes, electrostatic sensors, and an upper computer. Electrostatic sensors can convert the induced current into voltage signal output, which is convenient for subsequent signal transmission and processing. The response time of the electrostatic sensors is 1~2 milliseconds. The signal collected by the electrostatic sensors is transmitted to the upper computer through a wireless transmission device. The upper computer determines the information of the hand movement through the induced current signals and reflects it on the screen. At the same time, Leap Motion is used as the standard sensing system to verify the accuracy of the human hand movement sensing system, as shown in Figure 11. In practical application scenarios such as vehicle interactions, we think 50–350 mm is the proper operating range. If the hand is too close to the array, it may touch the electrodes. If the distance is too far, it can be considered that the hand exceeds the operation area and we can ignore it as interference.

**Figure 12:**
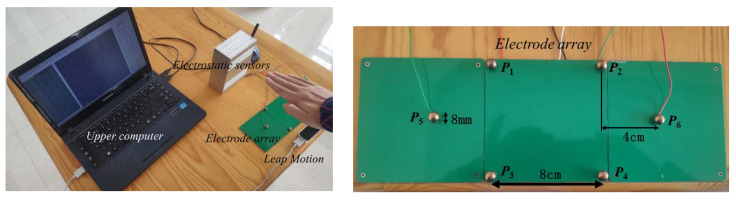
Experimental scene and electrode array.

In the experiment, the tester raised a hand and moved to 1 m in front of the electrode array. The sensing system calculates the height of a hand movement through the electrostatic signals obtained by the electrostatic sensors. Leap Motion and the electrode array are placed in the same plane so that they can simultaneously detect the height of the hand movement, as shown in the left section of Figure 12.

## 3. Results

**Figure 13:**
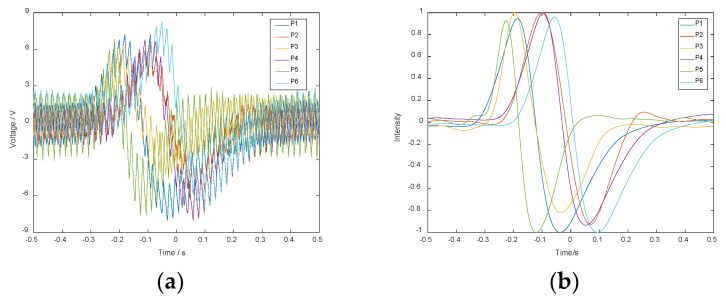
(**a**) Sensor output signals. (**b**) Filtered and normalized signals.

When the height of a hand movement is 97.41 mm given by Leap Motion, the output signals of the sensors are shown in Figure 13a. The values of filtered signals are normalized to −1~1, as shown in Figure 13b. And the measurement result of the system is 100.65 mm.

**Figure 14:**
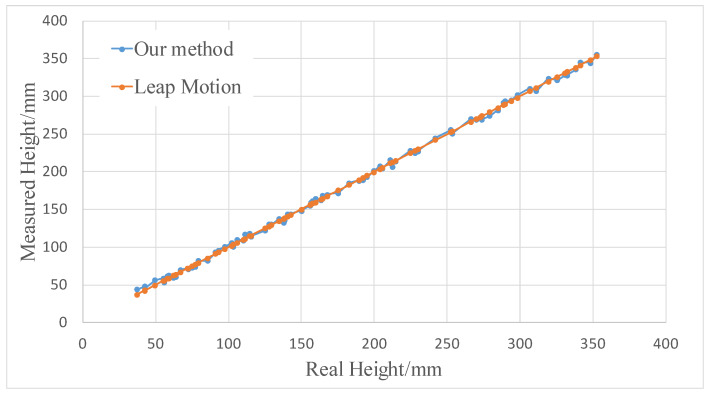
Comparison chart of the measurement results between the human hand movement sensing system and Leap Motion.

The upper computer calculated the height of the hand movement based on the received signals and compared it with the output values of Leap Motion. A total of 80 groups of data were collected at different heights, and the comparison results are shown in Figure 14.

**Figure 15:**
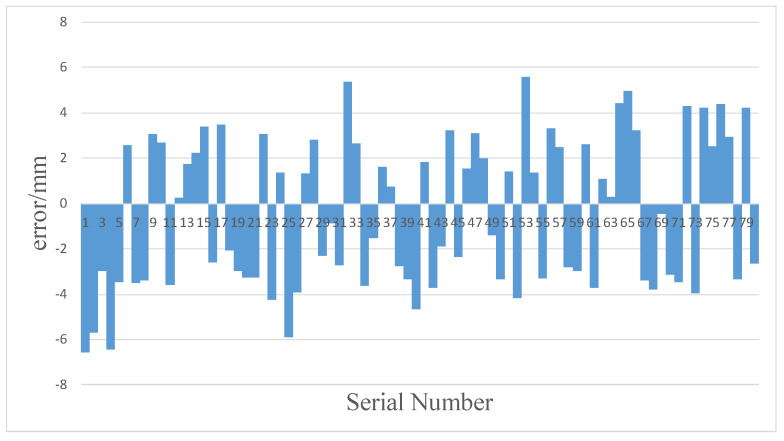
Statistical chart of the measurement errors of the human hand movement sensing system.

Figure 15 shows the errors of the results of the human hand movement sensing system. The maximum error is 6.58 mm and the mean error is 3.03 mm.

## 4. Discussion

### 4.1. Operation Error 

**Figure 16:**
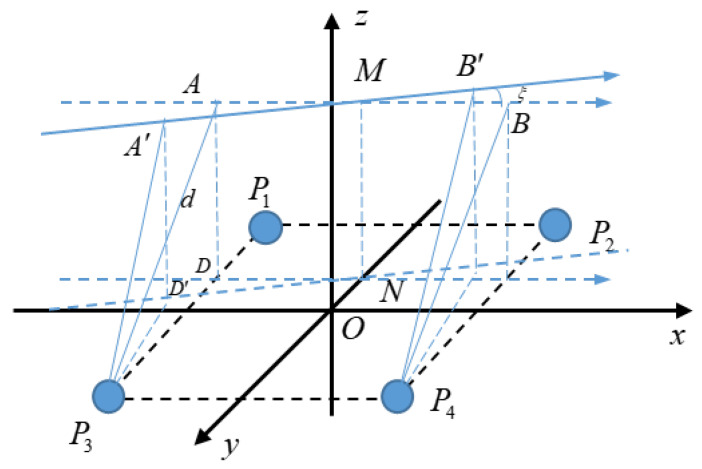
Schematic diagram of the height measurement error caused by the trajectory of a hand movement not completely parallel to the line of electrodes.

This paper proposes a method for measuring the height of hand movements based on a planar array of electrostatic induction electrodes. However, in actual human-computer interactions, it is impossible to ensure that the hand movement trajectory is completely parallel to the line of electrodes. Therefore, the measurement height inevitably has errors. The following will take direction ① as an example to analyze the errors. Assume that there is a deviation of *ξ* between the direction of the hand movement and direction ①, as shown in Figure 16. *AB* is the trajectory of the hand along direction ①, and *A’B’* is the trajectory of the hand with deviation angle *ξ*; *DN* and *D’N* are the projections of *AD* and *A’B’* on plane *XOY*; *AD* and *AD’* are perpendicular to plane *XOY,* respectively; and *d* is the distance from *AB* to line *P*_1_*P*_2_. The side length of the square *P*_1_*P*_2_*P*_3_*P*_4_ is 2*l*. Set the length of *P*_3_*D* as *m* and the length of *AD* as *h*.

When the hand moves along *A’B’*, the result solved by Equation (14) is:

(20) $$\begin{array}{ll} d+\Delta d & =D_{2}-\sqrt{2}L\frac{\Delta t_{2}}{\Delta t_{0}}\\ & =2\sqrt{2}l-2\sqrt{2}l\frac{\left(2l\cos\xi -\sqrt{{\left(l\tan\xi +m\right)}^{2}\cos^{2}\xi +h^{2}}/\tan\alpha_{2}\right)/v}{2l\cos\xi /v}.\\ & =2\sqrt{2}l-\frac{2\sqrt{2}l\cos\xi -\sqrt{{\left(l\tan\xi +m\right)}^{2}\cos^{2}\xi +h^{2}}}{\cos\xi } \end{array}$$

When the hand moves along direction ①, the distance *d* is:

(21) $$d=\sqrt{m^{2}+h^{2}}.$$

Then the absolute error *Δd* and the relative error *δ* of the measurement are, respectively:

(22) $$\left\{ \begin{array}{l} \Delta d=d+\Delta d-d\\ \delta =\frac{\Delta d}{d} \end{array}\right..$$

The measurement range of the system is 50~350 mm, *l* = 40 mm, and m∈[0,2l]. If the deviation angle *ξ* is less than 10°, then:

(23) $$\left\{ \begin{array}{l} \delta_{\max}=8.11\%\\ \Delta d_{\max}=6.88\mathrm{mm} \end{array}\right..$$

The maximum value of *δ* is obtained when *h* = 50 mm and *m* = 40 mm, and the maximum value of *Δd* is obtained when *h* = 350 mm and *m* = 80 mm.

In conclusion, when the angle deviation between the trajectory of the hand movement and the line of electrodes is within 10°, the measurement error can be ignored relative to the measuring range. The angle *ξ* can be calculated by the electrode array [26]. If the angle is larger than 10°, we can define it as a new gesture or think it is a wrong one and ignore it according to the situation.

### 4.2. Application Example

**Figure 17:**
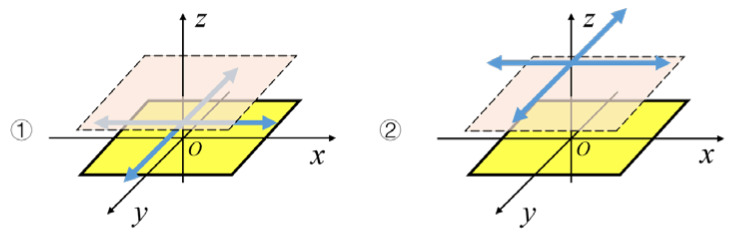
Schematic diagram of defined gestures.

In order to verify the usability of the method proposed in this paper, we wrote a program of a human-computer interface on the PC and obtained gesture information through the electrode array to control it. The area above the electrode array was divided into system operation area and application operation area. The system operation area was the area within 15 cm above the electrode array, and the gestures in this area were operations for controlling the system regardless of the current application, such as mute and standby. The application operation area was the area 15–30 cm above the electrode array, and gestures in this area were operations for controlling the current application or desktop. If the measured height of hand movement was more than 30 cm, it was considered as interference caused by distant charge movement (such as the hand movement of a person far away). The defined gestures are shown in Figure 17.

(1) In the system operation area, wave the hand to the right, and the system will initiate mute; wave the hand to the left, and the system will restore the original volume; wave the hand down, and the system will initiate standby; wave the hand up, and the system will be back to a normal state.

(2) In the application operation area, we can use gestures to operate the current application or desktop. Taking the desktop as an example, waving the hand in four directions moves the cursor to select different applications. Then we can enter the selected application with a gesture like tapping the screen.

**Figure 18:**
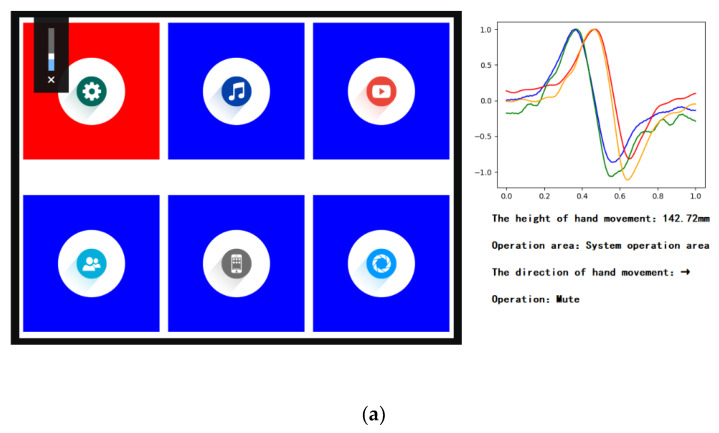

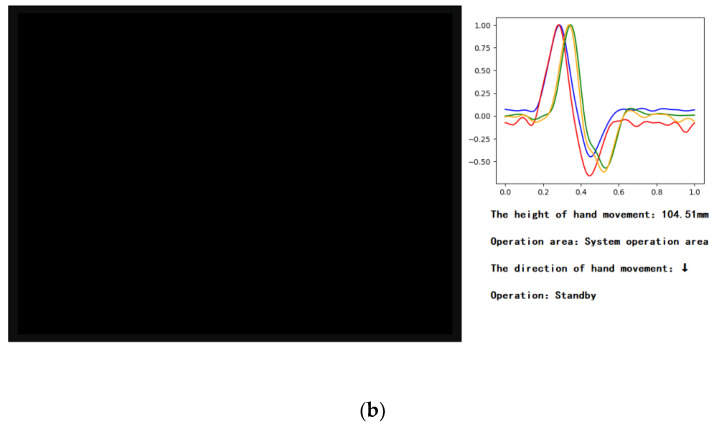
The interface reactions when the hand in the system operation area. (**a**) Waving the hand to the right; (**b**) waving the hand down.

This example only involves the movement of the hand in four directions, so only four electrodes are used. In the system operation area, when waving the hand to the right and down, the interface reactions are shown in Figure 18.

**Figure 19:**
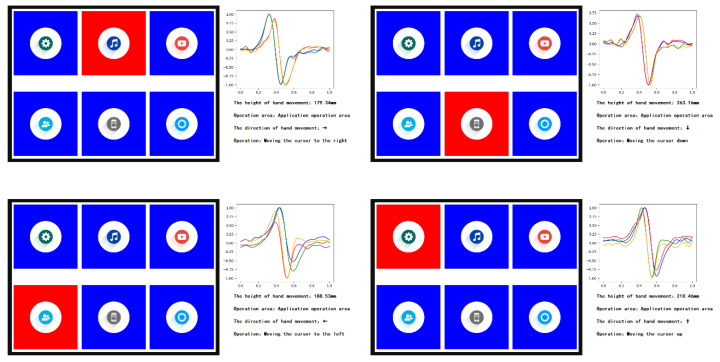
The interface reactions when waving the hand in four directions in the application operation area.

In the application operation area, when waving the hand in four directions, the interface reactions are shown in Figure 19.

**Figure 20:**
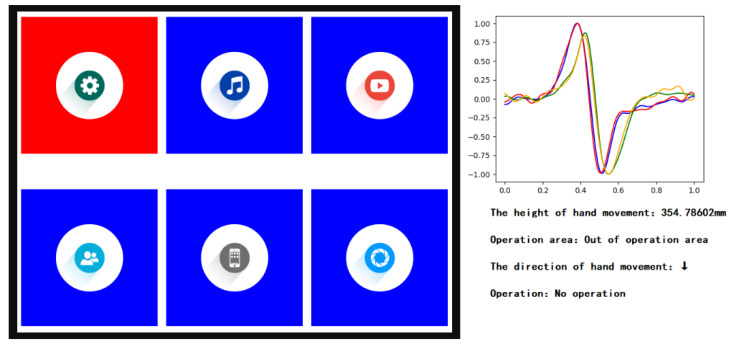
The interface reaction when the hand trajectory is out of operation area.

If the measured height of the hand movement was more than 30 cm, the interface reaction is shown in Figure 20.

In addition, we have found a way to measure the angle between the hand trajectory and the array when they are not parallel. And the gestures of the hand moving clockwise and counterclockwise can also be recognized. We will combine them with the method proposed in this article to enrich the gestures for human-computer interaction in our future research.

## 5. Conclusions

In order to obtain the height of hand movements based on a planar array of electrodes, a method for measuring the height of hand movements based on a planar array of electrostatic induction electrodes was proposed in this paper, and a human hand movement sensing system was established to verify it. The information of the induced current on the induced electrode as obtained by the electrostatic sensor, and was then converted into a voltage signal. The upper computer received signals from different electrodes to determine the direction and calculate the height of the hand movement. Finally, the results measured by the human hand movement sensing system were compared with those of Leap Motion set at the same time. It can be seen from the results that the system can measure the height of the hand movement with a maximum error of 6.58 mm. The system verifies that the algorithm can measure the height of the hand movement, and the measurement errors caused by the movement direction of the hand not being parallel to the line of electrodes were discussed.

The method proposed in this paper can be applied to human-computer interaction systems. After classifying the hand movement in eight directions of the plane, it can obtain the height of hand movements. This method has the characteristics of a simple algorithm, non-contact, and low power consumption. Compared with the methods proposed by Kai Tang, only a planar array of electrodes is needed, which greatly saves space and has a wider range of use. Compared with the vision-based system, this method does not need to place the sensors on the external surface of the equipment, so as to ensure the integrity of the external surface of the equipment and avoid the privacy problems caused by the camera and compared with wearable equipment, it is more convenient and comfortable to use.

We can use this method on an intelligent vehicle system to control some applications through gesture recognition. In this application scenario, vision-based interaction has complex algorithms and large power consumption, which will burden the vehicle’s power supply. In addition, the camera is easily affected by light and background, and it may cause privacy issues. The tridimensional array cannot be set up in the narrow environment of the vehicle. The method we propose can measure the height of hand movements using a planar array. Therefore, gestures of different heights can represent different operations. In addition, gestures outside the operating range can be ignored by the system as interference signals from the back row. Therefore, this method has a good application prospect.

## Figures and Tables

**Table 1:** Absolute values of the mean errors and the maximum errors under different SNR conditions.

SNR	10 dB		15 dB		20 dB	
**Absolute Values of Errors/mm**	**Mean Error**	**Max Error**	**Mean Error**	**Max Error**	**Mean Error**	**Max Error**
	13.32	28.71	5.04	11.70	1.09	5.76
